# Correlation of Aging and Segmental Choroidal Thickness Measurement using Swept Source Optical Coherence Tomography in Healthy Eyes

**DOI:** 10.1371/journal.pone.0144156

**Published:** 2015-12-03

**Authors:** Yu Wakatsuki, Ari Shinojima, Akiyuki Kawamura, Mitsuko Yuzawa

**Affiliations:** Department of Ophthalmology, School of Medicine, Nihon University, Tokyo, Japan; University of Sydney, AUSTRALIA

## Abstract

**Purpose:**

To assess and compare choroidal thickness changes related to aging, we determined whether changes are due to thinning of the choriocapillaris plus Sattler's (CS) layer and/or the large vessel layer in healthy eyes using swept-source optical coherence tomography (SS-OCT) at a wavelength of 1,050-nm.

**Methods:**

We studied 115 normal eyes of 115 healthy volunteers, all with refractive errors of less than -6 diopters. All 115 eyes underwent analysis of choroidal thickness at the fovea, the CS layer and the large choroidal vessel layer. In 68 of the 115 eyes, choroidal thickness was determined at five sites (the fovea, and superior, inferior, nasal, and temporal sites) using SS-OCT with an Early Treatment of Diabetic Retinopathy grid scan.

**Results:**

Total choroidal thicknesses at each of the five sites were related to subject age (P<0.0001). The choroid was thinnest at the nasal site, followed by the temporal, inferior, superior and finally the subfoveal site itself. The total choroidal thickness at the nasal site was significantly less than those at the other four sites (p<0.05). The CS layer showed thinning which correlated with age (P<0.0001). The thickness of the choroidal large vessel layer also decreased with age (p = 0.02). Subfoveal choroidal thickness was calculated as follows: 443.89–2.98×age (μm) (P<0.0001).

**Conclusion:**

Subfoveal choroidal thickness decreases by 2.98 μm each year. Total choroidal thickness diminishes with age. The CS and large vessel layers of the choroid at the subfovea showed significant decreases, though only the former correlated strongly with age.

## Introduction

The choroid contains a large number of blood vessels, pigments, and immune system components. The choroid absorbs light, supplies oxygen, and provides nourishment to protect the retina. However, the choroid is also the site of many pathological processes such as inflammation, ischemia and neovascularization. Optical coherence tomography (OCT), which became available in the late 1990s, allows non-invasive observations of the ocular fundus. Most recent ophthalmological studies have used OCT as a routine medical examination along with standard visual acuity testing. Time-domain OCT with an 820nm wavelength and 400 A-scans/sec represents a major advancement in the quality of OCT imaging as it allows better visualization of the retina. Spectral-domain (SD) OCT, providing in excess of 40,000 A-scans/sec, allows even more precise assessment of retinal structure, comparable to that of a histopathological specimen. Even SD-OCT is not, however, sufficient for detailed observations of the choroid.

Spaide et al. [1] reported the enhanced depth imaging (EDI)-OCT method based on SD-OCT, which enabled the chorioscleral border to be detected. However, clear choroidal images cannot be obtained in some cases, due to obscuring of the outer boundary of the choroid when the Heidelberg EDI-OCT device is employed [2,3]. Recently, swept-source (SS)-OCT with a wavelength of 1,050 nm and 100,000A-scans/sec has allowed in depth visualization of the eye from the retina to the sclera even in patients with moderate to severe cataracts, as well as during eye blinking and/or ocular movement. Therefore, SS-OCT provides clear, sharp images of the choroid. There are many reports describing overall choroidal thickness as decreasing with age [4–10], while no differences have been detected among sites in the healthy eye [5–10].

Branchini et al. [11] reported on the choroidal vasculature of healthy eyes, describing large choroidal vessel layer thickness and medium choroidal vessel layer/choriocapillaris layer thickness values obtained using spectral-domain OCT with an 840 nm wavelength. Esmaeelpour et al. [12] determined Sattler's and Haller's layer thicknesses using 3-dimensional 1060-nm OCT with an automatic measurement function. However, their method was used without determining the border between Sattler’s and Haller’s layers and may not be feasible. In our view, only manual measurements allow Sattler's and Haller's layers to be clearly distinguished.

Neither of these prior reports examined, or even took into consideration, the age-change correlation with choroidal thickness. Therefore, using SS-OCT, we investigated choroidal thickness (choriocapillaris plus Sattler's (CS) layer and/or large vessel layer) in relation to age. We manually measured layer thicknesses, to obtain clear images and to derive formulas that would allow us to calculate thickness based on OCT data.

We assessed choroidal thinning using SS-OCT manually to assure clear measurements, in relation to age decades, in order to determine whether the CS and/or the large vessel layer contributes to the widely recognized decrease in the thickness of the choroidal layer with advancing age. We also endeavored to clarify the etiology of choroidal thinning.

## Materials and Methods

Between September 2013 and August 2014, healthy patients were recruited for this study. All subjects were at least 21 years of age and had normal eyes. None had visual symptoms or a history of ocular disease. The only inclusion criterion was refractive error less than -6 diopters (D). This criterion was applied to eliminate the potential effects of axial length and high myopia [13]. Participant details are presented in Tables 1 and 2.

**Table 1:** Age and number of eyes by age decade at each site.

Age decade (years)	Mean age (years)	Number of eyes (subjects)
21–29	26.3±3.1	9 (9)
30–39	35.4±3.7	9 (9)
40–49	44.2±2.8	18 (18)
50–59	54.6±2.7	18 (18)
60–69	64.4±2.8	28 (28)
70–79	73.4±3.0	24 (24)
80–85	81.2±1.7	9 (9)

**Table 2:** Age and number of eyes by age decade in each area.

Age decade (years)	Mean age (years)	Number of eyes (subjects)
21–29	26.3±3.1	9 (9)
30–39	35.2±1.9	6 (6)
40–49	44.1±3.1	10 (10)
50–59	54.4±2.6	11 (11)
60–69	63.6±2.5	14 (14)
70–79	73.5±3.2	12 (12)
80–85	81.5±2.1	6 (6)

The institutional review board of Nihon University Hospital, Tokyo, Japan, deemed approval to be unnecessary for this study (Waiver of approval the consent process form). For this study, we recruited volunteers, who visited our hospital to undergo periodic eye examinations, obtaining verbal consent without a signature in all cases. Furthermore, all participants had the right to opt-out of the study. If the volunteers agreed to participate in this study, they did not need to sign a consent form. If a volunteer decided to drop out of the study, we requested that they sign a consent form to opt-out of the study.

We chose 115 eyes (62 males, 53 females) for analysis of the thickness of each subfoveal layer (the CS layer and the large vessel layer) as the primary endpoint. Out of these 115 eyes, we chose 68 (34 males, 34 females) eyes for subgroup analysis of choroidal thickness in five areas (at the fovea itself, and at the sites superior, inferior, nasal, and temporal to the fovea).

We examined only one eye, mainly the right eye, in each subject to eliminate individual variation. However, if the right met any of our exclusion criteria, we studied the left eye instead.

It is necessary to measure all 512 (horizontal) and 64 (vertical) lines clearly when measuring mean choroidal thicknesses of the five aforementioned areas. If even one slice is missing, due to blinking, the mean choroidal thickness value will not be accurate. Therefore, five of the 68 eyes were excluded from the analysis of choroidal thickness due to blinking during the examination. There are no effects of blinking on choroidal thickness at the fovea.

We performed horizontal and axial scans and three-dimensional analysis of a 6 by 6 mm area at the center of the fovea using SS-OCT (Topcon Corp., Tokyo, Japan). The three-dimensional images were obtained with SS-OCT using raster scans of 512 (horizontal) and 64 (vertical) lines. The A-scan value per dataset (total 32,768 axial scans) was 0.8 seconds. Choroidal thickness and the chorioscleral border were measured manually with a built-in caliper (Y.W.), according to Rhaman et al [14]. In each case, choroidal thickness was measured from the bottom of the hyper-reflective line of the retinal pigment epithelium (RPE) to the chorioscleral border.

**Fig 1:**
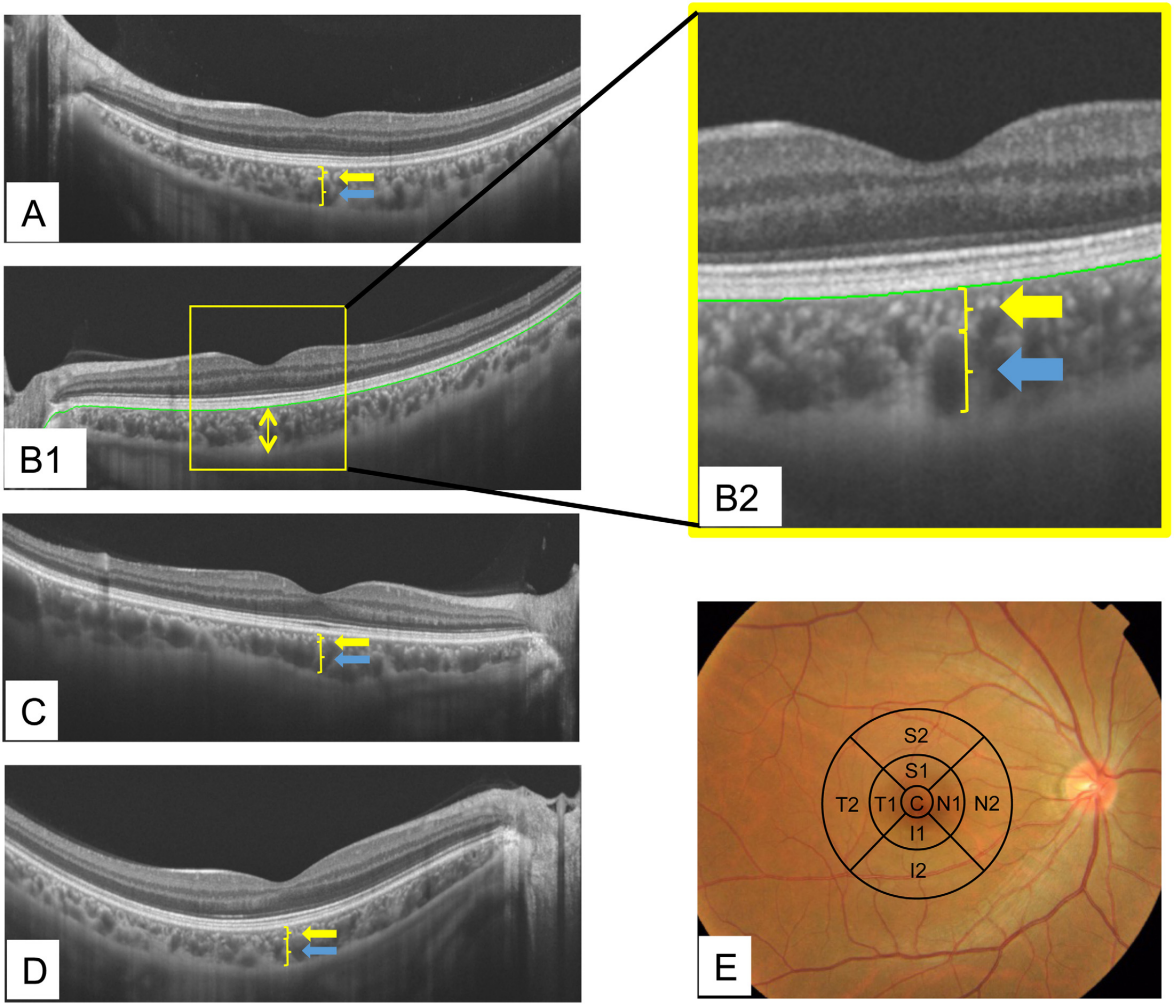
The choriocapillaris plus Sattler’s (CS) layer and the choroidal large vessel layer. (A-D) Choroidal thickness of each layer. The bold yellow arrow indicates the choriocapillaris plus Sattler’s layer, the bold blue arrow the choroidal large vessel layer. Total subfoveal choroidal thickness is the sum of these two layers (between the yellow and blue arrows). (B1) The green line represents the bottom of the RPE, the two yellow arrows the thickness of the subfoveal choroid. (B2) This image is the expanded image of the yellow square portion of B1. (E) Early Treatment Diabetic Retinopathy Study (ETDRS) macular map sectors. The central area represents the fovea. Mean macular thickness was calculated for the superior (S), inferior (I), temporal (T), and nasal (N) areas, all for diameters of 1 mm, 3 mm, and 6 mm. We defined ‘C’ as the “mean foveal choroidal thickness area”, and each of the other four areas as follows: ‘S1+S2 = mean superior choroidal thickness area’, ‘I1+I2 = mean inferior choroidal thickness area’, ‘N1+N2 = mean nasal choroidal thickness area’, and ‘T1+T2 = mean temporal choroidal thickness area’.

Choroidal thickness was measured at the fovea and at each of the other four sites (superior, inferior, nasal, and temporal to the fovea) 3mm from the fovea for each age decade employing the Early Treatment Diabetic Retinopathy Study (ETDRS) grid (Fig 1). The mean choroidal thickness within the ETDRS grid was averaged by SS-OCT automatically. The ETDRS grid consists of three circles; the outermost circle has a radius of 3 mm, the second circle of 1.5 mm, and the innermost circle of 0.5 mm.

We defined “site” as a pin-point portion of the retina and used the term “area” for the broader portion. We defined the superior area as the total of the outer and inner superior areas as well as those of the inferior, nasal, and temporal areas (Fig 1). The average choroidal thickness (6,872mm^2^, 6,255 measured points) was calculated for each of the four areas. The foveal area was taken to be that surrounded by the innermost circle (0.785mm^2^, 715 measured values) and its average thickness was calculated.

Chan-Ling et al. reported that the undifferentiated human’s capillary plexus forms first and that the layers of larger vessels form thereafter [15], and calponin was expressed only on large vessels [16]. From these reports, based on immunohistochemical observations, we can reasonably speculate that there are differences between the CS and large choroidal vessel layers. Therefore, we investigated the CS and large choroidal layers. We defined the CS layer as starting just below the RPE line and extending to the upper border of the hypo-reflective large tubular structure area (choroidal large vessel layer), as in a previous report (Fig 1) [11].

### Items investigated

The following items were investigated; subfoveal choroidal thickness and the choroidal thicknesses at the aforementioned four sites 3mm from the fovea for each age decade and each eye, as well as the mean choroidal thickness at each of these five areas. The thicknesses of the subfoveal CS layer and the large vessel layer were also determined (Fig 1).

### Statistical Analysis

The relationships between age and choroidal thicknesses were evaluated using regression models and regression coefficients. For the comparisons among choroidal thicknesses adjusted for age, a mixed model was applied, and we evaluated the differences of choroidal thickness and the distance between regression lines. The statistical power was 0.493, which was calculated based on a previous report, employing a distance of ‘23 μm’ for our 68 subjects (significance level of 0.05) [14]. We defined statistically significant differences as having a value of p<0.05. SAS version 9.3 was used for all statistical analyses.

## Results

**Fig 2:**
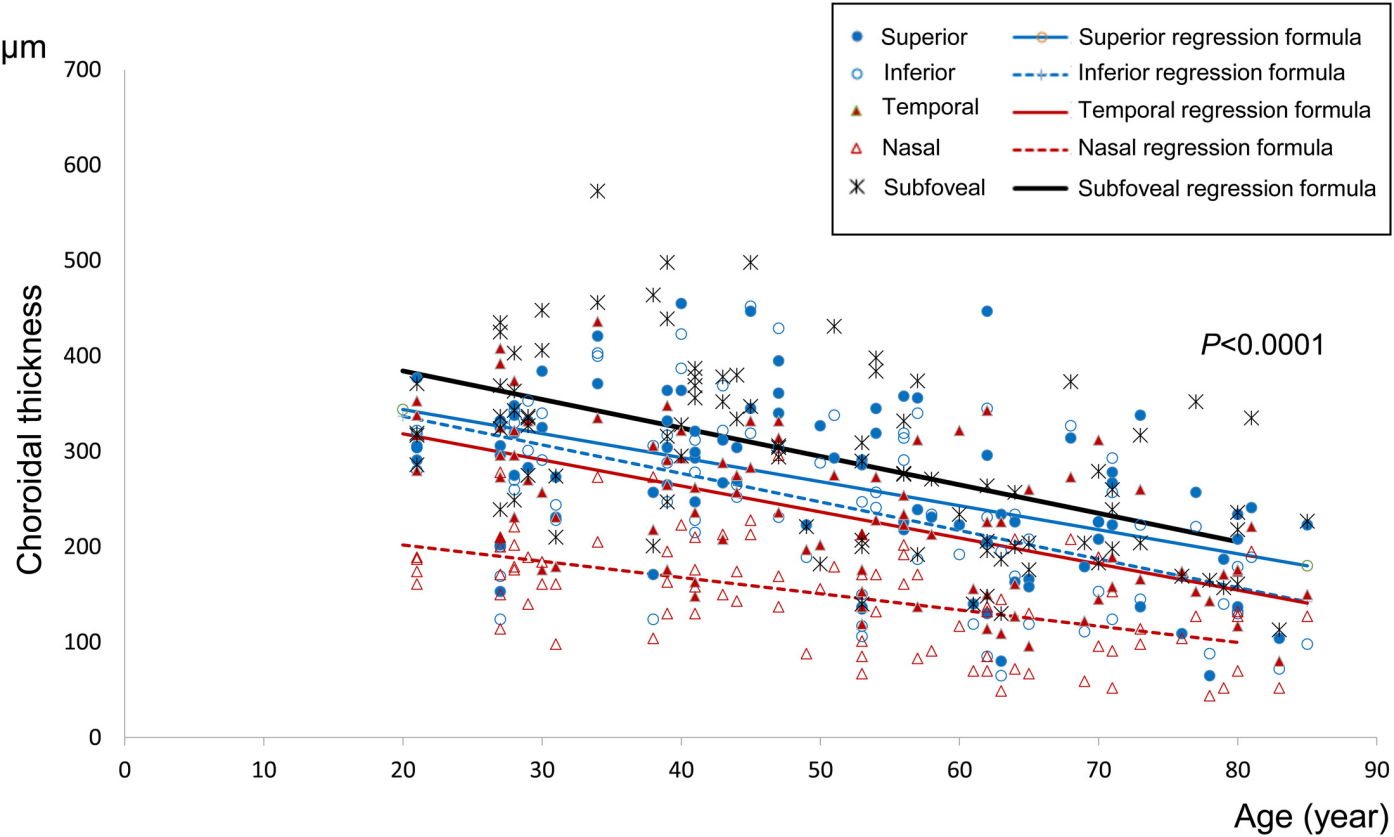
Choroidal thicknesses at five measurement sites by age. Scatterplot for age and choroidal thickness at each site, as measured at a single point, are shown. There is a negative correlation between age and choroidal thickness, and the nasal site of the choroid appears to be the thinnest, followed by the temporal, inferior, superior, and foveal sites.

The subfoveal choroidal thickness and those at the four sites 3mm from the fovea are shown in Table 3 and Fig 2.

**Table 3:** Mean choroidal thicknesses at each of the five sites.

Age	No. of eyes	Subfoveal Site	Superior Site	Inferior Site	Nasal Site	Temporal Site
20s	9	336.33±52.38	300.22±50.79	289.50±62.61	181.67±35.17	312.17±50.80
30s	9	377.67±117.86	308.17±68.86	286.25±73.83	178.50±53.52	268.25±73.82
40s	18	345.44±57.78	327.69±63.84	310.94±78.98	183.19±48.81	259.13±55.64
50s	18	286.88±83.01	259.87±76.08	237.60±78.12	145.00±46.33	209.93±54.79
60s	28	208.50±59.43	211.50±89.20	185.93±80.23	110.64±50.38	191.79±79.52
70s	24	230.62±69.57	208.64±76.25	185.36±59.77	101.82±42.08	184.45±50.71
80s	9	215.00±68.69	191.17±51.88	133.33±41.25	117.17±46.45	153.00±45.14
Total	115					

### Mean choroidal thicknesses of each of the five sites (Table 3)

#### Subfoveal choroidal thickness

Mean choroidal thickness values (±SD) for each age decade were; 20s: 336.33±52.38 μm, 30s: 377.67±117.86 μm, 40s: 345.44±57.78 μm, 50s: 286.88±83.01 μm, 60s: 208.50±59.43 μm, 70s: 230.62±69.57 μm, and 80s: 215.00±68.69 μm.

These data confirm a significant linear correlation between subfoveal choroidal thickness and age (p<0.0001). The regression formula is: macular choroidal thickness (μm) = 443.89–2.98×age (p<0.0001). Mean subfoveal choroidal thickness decreased by 2.98 μm per year.

#### Superior site

Mean choroidal thickness values (±SD) at the site superior to the fovea for each age decade were; 20s: 300.22±50.79 μm, 30s: 308.17±68.86 μm, 40s: 327.69±63.84 μm, 50s: 259.87±76.08 μm, 60s: 211.50±89.20 μm, 70s: 208.64±76.25 μm, and 80s: 191.17±51.88 μm.

There was a linear relationship between superior site choroidal thickness and age. The regression formula is: superior choroidal thickness (μm) = 396.36–2.52×age (p<0.0001). Mean choroidal thickness at the superior site decreased by 2.52 μm per year.

#### Inferior site

Mean choroidal thickness values (±SD) at the site inferior to the fovea for each age decade were; 20s: 289.50±62.61 μm, 30s: 286.25±73.83 μm, 40s: 310.94±78.98 μm, 50s: 237.60±78.12 μm, 60s: 185.93±80.23 μm, 70s: 185.36±59.77 μm, and 80s: 133.33±41.25 μm.

There was a linear relationship between choroidal thickness at the inferior site and age. The regression formula is: inferior choroidal thickness (μm) = 396.66–2.99×age (p<0.0001). Mean choroidal thickness at the inferior site decreased by 2.99 μm per year.

#### Nasal site

Mean choroidal thickness values (±SD) at the nasal site for each age decade were; 20s: 181.67±35.17 μm, 30s: 178.50±53.52 μm, 40s: 183.19±48.81 μm, 50s: 145.00±46.33 μm, 60s: 110.64±50.38 μm, 70s: 101.82±42.08 μm, and 80s: 117.17±46.45 μm.

There was a linear relationship between choroidal thickness at the nasal site and age. The regression formula is: nasal choroidal thickness (μm) = 236.01–1.70×age (p<0.0001). Mean choroidal thickness at the nasal site decreased by 1.70 μm per year.

#### Temporal site

Mean choroidal thickness values (±SD) at the temporal site for each age decade were; 20s: 312.17±50.80 μm, 30s: 268.25±73.82 μm, 40s: 259.13±55.64 μm, 50s: 209.93±54.79 μm, 60s: 191.79±79.52 μm, 70s: 184.45±50.71 μm, and 80s: 153.00±45.14 μm.

There was a linear relationship between choroidal thickness at the temporal site and age. The regression formula is: temporal choroidal thickness (μm) = 372.95–2.73×age (p<0.0001). Mean choroidal thickness at the temporal site decreased by 2.73 μm per year.

The choroid was thinnest at the nasal site, followed in order by the temporal, inferior, superior and subfoveal sites. There was no significant difference in choroidal thickness between the temporal and inferior sites (p = 0.19) for any of the age groups (Table 4).

**Table 4:** Correlations among the mean choroidal thicknesses of the five sites.

Site×Site	Difference estimate	Standard error	Variance	t-value	Pr >t	R^2^
Superior×Inferior	21.95	7.17	391	3.06	0.0023	0.51
Superior×Temporal	31.33	7.17	391	4.37	<.0001	0.58
Superior×Nasal	116.91	7.17	391	16.32	<.0001	0.61
Superior×Foveal	-27.37	7.17	391	-3.84	0.0001	0.58
Inferior×Temporal	9.38	7.17	391	1.31	0.19	0.54
Inferior×Nasal	94.97	7.17	391	13.25	<.0001	0.62
Inferior×Foveal	-49.31	7.17	391	-6.92	<.0001	0.59
Temporal×Nasal	85.59	7.17	391	11.94	<.0001	0.48
Temporal×Foveal	-58.699	7.17	391	-8.24	<.0001	0.56
Nasal×Foveal	-144.28	7.17	391	-20.25	<.0001	0.63

### Mean choroidal thicknesses of each of the five areas (Table 5)

**Table 5:** Mean choroidal thicknesses of each of the five areas.

Age	No. of eyes	Foveal area	Superior area	Inferior area	Nasal area	Temporal area
20s	9	296.44±43.56	300.44±35.67	286.11±58.33	254.33±37.89	309.11±36.70
30s	6	295.17±111.87	277.42±89.60	289.58±104.49	256.25±95.87	288.50±101.75
40s	10	306.50±69.36	300.75±48.45	302.35±79.85	259.25±61.84	278.75±52.78
50s	11	234.55±85.77	246.18±75.39	236.82±73.33	218.59±64.90	217.23±74.28
60s	14	187.64±57.97	179.91±55.65	189.59±59.53	154.23±56.46	184.95±53.66
70s	12	206.73±65.38	208.91±70.85	194.73±59.70	168.64±56.91	205.14±50.72
80s	6	204.80±57.10	213.20±57.84	168.90±46.24	167.50±53.18	182.90±41.87
Total	68					

#### Foveal area (innermost circle area)

Mean choroidal thickness values (±SD) for each age decade were; 20s: 296.44±43.56 μm, 30s: 295.17±111.87 μm, 40s: 306.50±69.36 μm, 50s: 234.55±85.77 μm, 60s: 187.64±57.97 μm, 70s: 206.73±65.38 μm, and 80s: 204.80±57.10 μm.

There was a linear relationship between mean choroidal thickness in the foveal area and age. The regression formula is: mean foveal choroidal thickness (μm) = 366.27–2.24×age (p<0.0001). Mean choroidal thickness in the foveal area decreased by 2.24 μm per year.

#### Superior area

Mean choroidal thickness (±SD) values for each age decade were; 20s: 300.44±35.67 μm, 30s: 277.42±89.60 μm, 40s: 300.75±48.45 μm, 50s: 246.18±75.39 μm, 60s: 179.91±55.65 μm, 70s: 208.91±70.85 μm, and 80s: 213.20±57.84 μm.

There was a linear relationship between mean choroidal thickness in the superior area and age. The regression formula is: mean superior choroidal thickness (μm) = 364.66–2.22×age (p<0.0001). Mean choroidal thickness in the superior area decreased by 2.22 μm per year.

#### Inferior area

Mean choroidal thickness values (±SD) for each age decade were; 20s: 286.11±58.33 μm, 30s: 289.58±104.49 μm, 40s: 302.35±79.85 μm, 50s: 236.82±73.33 μm, 60s: 189.59±59.53 μm, 70s: 194.73±59.70 μm, and 80s: 168.90±46.24 μm.

There was a linear relationship between mean choroidal thickness in the inferior area and age. The regression formula is: mean inferior choroidal thickness (μm) = 373.99–2.51×age (p<0.0001). Mean choroidal thickness in the inferior area decreased by 2.51 μm per year.

#### Nasal area

Mean average choroidal thickness values (±SD) for each age decade were; 20s: 254.33±37.89 μm, 30s: 256.25±95.87 μm, 40s: 259.25±61.84 μm, 50s: 218.59±64.90 μm, 60s: 154.23±56.46 μm, 70s: 168.64±56.91 μm, and 80s: 167.50±53.18 μm.

There was a linear relationship between mean choroidal thickness in the nasal area and age. The regression formula is: mean nasal choroidal thickness (μm) = 325.52–2.14×age (p<0.0001). Mean choroidal thickness in the nasal area decreased by 2.14 μm per year.

#### Temporal area

Mean choroidal thickness values (±SD) for each age decade were; 20s: 309.11±36.70 μm, 30s: 288.50±101.75 μm, 40s: 278.75±52.78 μm, 50s: 217.23±74.28 μm, 60s: 184.95±53.66 μm, 70s: 205.14±50.72 μm, and 80s: 182.90±41.87 μm.

There was a linear relationship between mean choroidal thickness in the temporal area and age. The regression formula is: mean temporal choroidal thickness (μm) = 369.69–2.47×age (p<0.0001). Mean choroidal thickness in the temporal area decreased by 2.47 μm per year.

**Fig 3:**
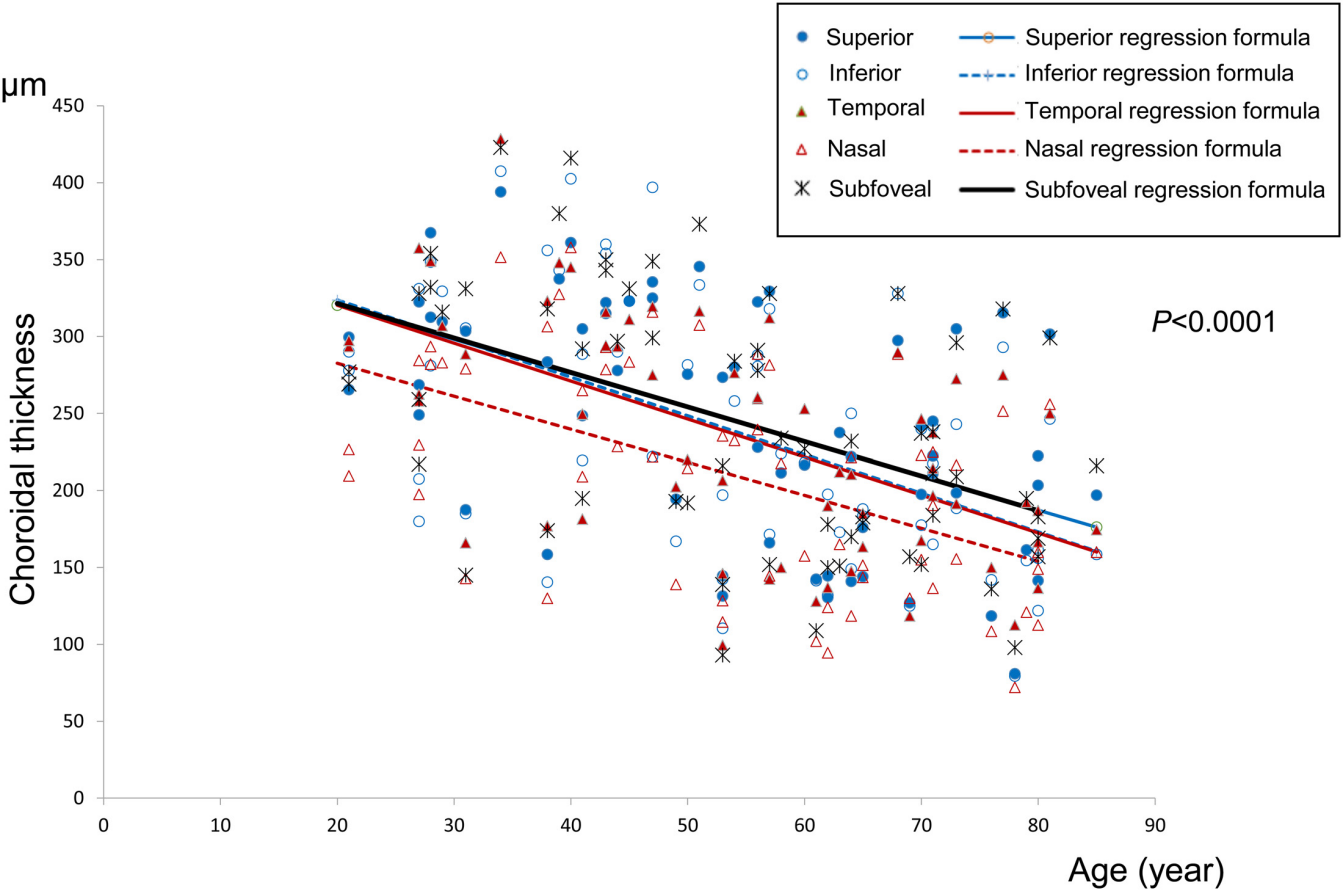
Mean choroidal thickness at each site by age. Scatterplot of age and the mean choroidal thickness, by sector, at each measurement site are shown. There is a negative correlation between age and mean choroidal thickness. The choroid appears to be thinnest at the nasal site.

The choroid was significantly thinner in the nasal area than in the other four areas (p<0.05), while there were no significant differences among the other four areas (p>0.05) (Table 6 and Fig 3).

**Table 6:** Correlations of mean choroidal thickness values between each pair of areas.

Area×Area	Difference estimate	Standard error	Variance	t-value	Pr >t	R^2^
Superior×Inferior	6.57	11.76	309	0.56	0.58	0.27
Superior×Temporal	8.44	11.76	309	0.72	0.47	0.29
Superior×Nasal	35.15	11.76	309	2.99	0.003	0.34
Superior×Foveal	-0.3	11.76	309	-0.03	0.98	0.27
Inferior×Temporal	1.87	11.76	309	0.16	0.87	0.23
Inferior×Nasal	28.58	11.76	309	2.43	0.016	0.27
Inferior×Foveal	-6.87	11.76	309	-0.58	0.56	0.29
Temporal×Nasal	26.71	11.76	309	2.27	0.024	0.34
Temporal×Foveal	-8.74	11.76	309	-0.74	0.46	0.27
Nasal×Foveal	-35.45	11.76	309	-3.01	0.0028	0.23

### Thicknesses of the subfoveal CS layer and the large vessel layer

#### Subfoveal CS layer thickness by age

Mean CS layer thickness values (±SD) for each age decade were; 20s: 110.78±40.80 μm, 30s: 89.89±25.16 μm, 40s: 73.11±28.25 μm, 50s: 54.56±23.03 μm, 60s: 50.57±29.06 μm, 70s: 55.42±38.37 μm, and 80s: 39.89±32.47 μm.

**Fig 4:**
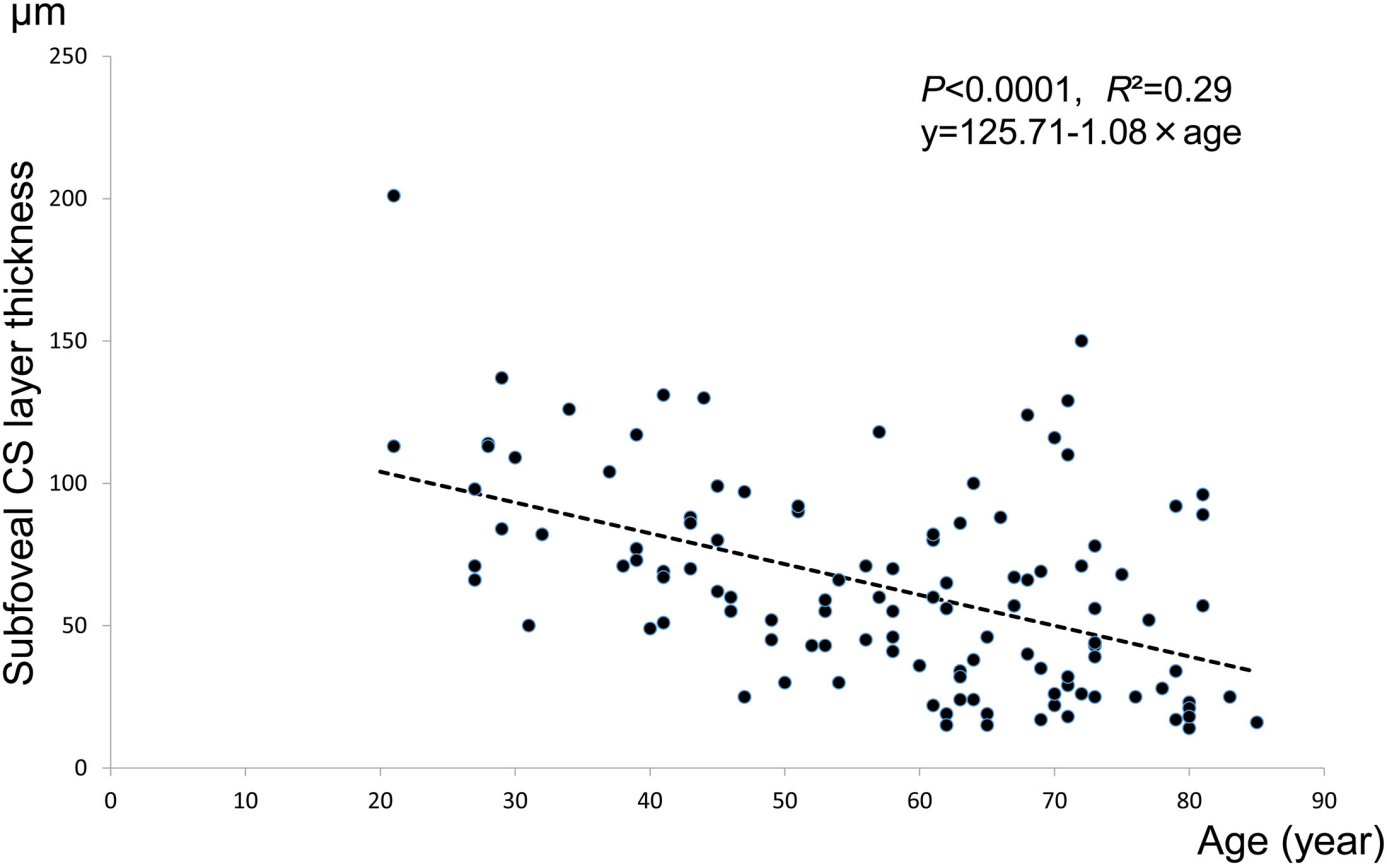
Subfoveal CS layer thickness. Scatterplot of age and subfoveal CS layer choroidal thickness, measured at a single point, are presented. There is a negative correlation between age and the choroidal thickness of the CS layer (p<0.0001, y = 125.71–1.08×age. R^2^ = 0.29).

There was a linear relationship between CS layer thickness and age (Fig 4). The regression formula is: CS layer thickness (μm) = 125.71–1.08×age (p<0.0001, R^2^ = 0.29).

#### Subfoveal choroidal large vessel layer thickness by age

Mean subfoveal choroidal thickness values (±SD) of the large vessel layer for each age decade were; 20s: 214.56±74.54 μm, 30s: 239.5±88.86 μm, 40s: 225.59±47.75 μm, 50s: 183.40±55.08 μm, 60s: 185.86±58.76 μm, 70s: 189.26±49.82 μm, and 80s: 200.00±48.53 μm.

**Fig 5:**
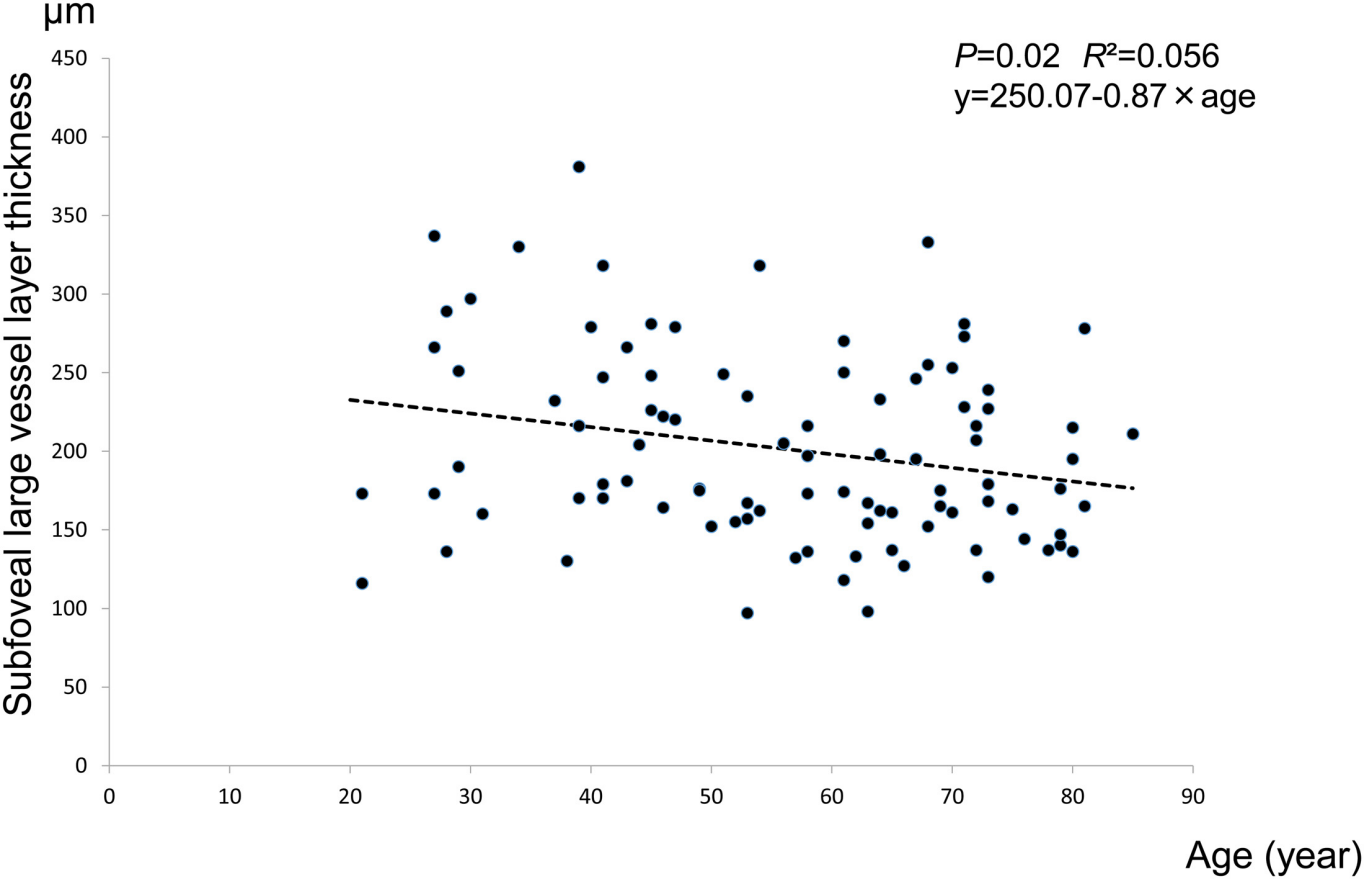
Subfoveal large vessel layer thickness. Scatterplot for age and subfoveal large vessel layer choroidal thickness measured at a single point are presented. There is a negative correlation between age and the choroidal thickness of the large vessel layer (p = 0.0209, y = 250.07–0.87×age. R^2^ = 0.056).

There was a linear relationship between large vessel layer thickness and age (Fig 5). The regression formula is: choroidal large vessel layer thickness (μm) = 250.07–0.87×age (p<0.0209, R^2^ = 0.056). A significant age-dependent difference in choroidal thickness was observed for both the CS layer (p<0.0001) and the large vessel layer (p<0.0209) (Table 7, Figs 4 and 5).

**Table 7:** CS layer and large vessel layer choroidal thickness by age decade.

Age	No. of Eyes	Subfoveal CS layer	Subfoveal choroidal large vessel layer
20s	9	110.78±40.80	214.56±74.54
30s	9	89.89±25.16	239.5±88.86
40s	18	73.11±28.25	225.59±47.75
50s	18	54.56±23.03	183.40±55.08
60s	28	50.57±29.06	185.86±58.76
70s	24	55.42±38.37	189.26±49.82
80s	9	39.89±32.47	200.00±48.53
Total	115		

The statistical power was 0.493, which was calculated based on a previous report, employing a distance of ‘23 μm’ for our 68 subjects (significance level of 0.05).[14]

## Discussion

Chen et al reported axial length to be the main predictor of foveal choroidal thickness but they did not examine refractive error [17]. High myopia may reportedly affect subfoveal choroidal thickness in Japanese eyes, a factor significantly associated with refractive error [18,19]. Refractive error was, however, reported to correlate significantly with posterior staphyloma height [19]. Fujiwara et al. reported a correlation between subfoveal choroidal thickness and refractive error, and noted that subfoveal choroidal thickness tended to correlate negatively with refractive error, though this correlation was not significant between -6D and +6D. (p = 0.10, R^2^ = 0.1) [13].

Therefore, high myopia -6D or greater was excluded in our present study to eliminate the effect of axial length and high myopia. Since lymphatics are also highly related to axial length changes in myopia, we need to consider this factor in future studies [20].

The subfoveal choroidal thickness regression formula yielded differences ranging from 2.98 μm (mean subfoveal choroidal thickness) to 2.24 μm (mean choroidal thickness at the foveal area).

There are many reports describing choroidal thickness in each of the aforementioned areas, i.e. the fovea itself, and the sites superior, inferior, nasal, and temporal to the fovea [5–10].

The choroidal thicknesses of both sites and areas decreased with age. The choroid was thinnest at the nasal site, followed by the temporal, inferior, superior, and foveal sites. The choroid was significantly thinner at the nasal site than at the other four sites. Choroidal thickness averages for each site were derived from a broad area extending from the outer portion of the choroid to the fovea itself, but the choroidal thicknesses of each site were obtained from only a single measurement. Therefore, a lack of measurement data might have produced differences.

There are no reports, to our knowledge, presenting a formula relating the thicknesses of distinct parts of the choroid with age. The correlations between mean choroidal thicknesses and age are presented as scatter diagrams with minor deflection, with p values for degree of skewing and the intercept being <0.0001. We thus consider our data to be reliable.

Esmaeelpour et al reported automatic measurements of the thicknesses of Sattler's layer and Haller's layer at the choroid using 3-dimensional OCT with a 1060-nm wavelength [12]. We manually measured CS and large choroidal vessel layer thicknesses using SS-OCT, encountering no difficulties such as obscuring of the border between the layers.

We endeavored to assess choroidal thickness related to age decades and to determine which layer(s) might be involved in the observed choroidal thinning. Therefore, we conducted manual segmentation, employing a built-in caliper, and analyzed the thickness of both the CS layer and the choroidal large vessel layer. The thickness of the subfoveal CS layer decreased with age and the correlation was statistically significant (p<0.0001). The thickness of the large vessel layer also decreased with age (p = 0.0204), indicating a trend for the thickness of this layer to decrease with aging. The p values indicated, however, that the decrease in the CS layer was more than 200 times that in the large choroidal vessel layer. Therefore, we speculate that the aging change in total choroidal thickness at the fovea is mainly attributable to a decrease in CS layer thickness. The resolution quality of SS-OCT is not sufficient to allow the choriocapillaris layer to be distinguished from Sattler’s layer. In addition, the choriocapillaris is too thin to be evaluated by itself.

In this study, we were also interested in vascular endothelial growth factor (VEGF)-A, which is secreted by the RPE and exerts a trophic influence on the choriocapillaris. The RPE secretes VEGF toward its basal side, where VEGF receptors are located on the adjacent choriocapillaris endothelium, suggesting VEGF to play a role in the maintenance of choriocapillaris fenestration in the normal eye [21,22]. An increased lipid content, documented in Bruch's membrane with aging in some cases, has also been postulated to specifically impede the movement of water and water-soluble agents between the choroidal and RPE compartments [23,24]. Furthermore, lipid accumulation appears to be higher in the macula than in the peripheral fundus [21,25,26].

It has been suggested that in hypoxic states and with aging, this paracrine relationship is disturbed by a thickened Bruch’s membrane, and that this age-associated change may block the passage of VEGF-A from the RPE to the choriocapillaris [21,26,27]. In normal eyes, as revealed by autopsy but not studies of the eyes of living subjects, from the first to the tenth decade of life the choriocapillaris diameter and choroidal thickness decrease while the thickness of Bruch’s membrane increases with age [4].

Our results reflect the choroidal layer changing with age, possibly in relation to the functions of VEGF-A and lipid accumulation. The CS layer is closer to the RPE layer than to the choroidal large vessel layer, i.e., the CS layer would presumably be more affected by the aging changes described in prior reports. However, the CS layer consists of Sattler’s layer and the choriocapillaris layer. Whether one or both of these layers contributes to diminished choroidal thickness with aging remains as yet unknown.

We speculate that not only VEGF but also systemic water may exert effects on the thicknesses of ocular layers. The total amount of water in the body decreases with age, possibly contributing to the observed decreases in choroidal thickness with aging. The water drinking test revealed that as the amount of water in the body increases, choroidal thickness also increases [28]. Although VEGF might significantly influence differences between the thicknesses of the large choroidal vessel and CS layers, the origin of such differences is unknown.

## Conclusions

We found that subfoveal choroidal thickness decreases by 2.98 μm per year, on average. Choroidal thickness decreased at all measurement sites with age. Thicknesses of the subfoveal CS layer and the large vessel layer decreased significantly, and that of the CS layer correlated especially strongly with age.
